# Supplementary material for: Participatory monitoring and evaluation approaches that influence decision-making: lessons from a maternal and newborn study in Eastern Uganda
Source: Health Res Policy Syst. 2017 Dec 28;15(Suppl 2):107. doi: 10.1186/s12961-017-0274-9 (PMC5751403; doi:10.1186/s12961-017-0274-9)
Supplement: Supplementary file 1 — Changes in savings and transport for health. (DOC 31 kb) [file 12961_2017_274_MOESM1_ESM.doc]

*“Nowadays they [savings groups] have boda-bodas. For example, my savings group in my sub county (Kabweri), they have specific boda-bodas in each saving group that does the transportation of these women to the health facility to access services to avoid maternal and new born deaths. This engagement began with the coming of the MANIFEST project, you know at first, people used to save, but the saving they used to have was for Christmas day, for buying cows for their meat and other things. These days mostly people save money unlike those days when they used to save for Christmas for buying goats, cows, they didn’t have that knowledge of saving to prepare for birth. But with the coming of MANIFEST to pass on that information people have realized that it’s important to save. Apart from giving birth, there are other issues that need money now people have realized. They know how to make money in order to save. That is a very good benefit from manifest project.*

*And the issue of transportation is very good because it saves lives, so me people in the villages, you know what the village is!! Time comes for birth she has no money in the pocket, even the bed sheets, no razor blade, no soap, but now when these women are engaged in a group, they talk to them to prepare to be ready because giving birth will never tell that today or tomorrow I will come. So at least that saved money should be there for you (woman). These transporters are paid after they [boda-bodas] have transported our own women to the health facility through their savings unlike those days when they used to deliver in banana plantations.*

*Even in our health facilities these days, they know that “manifest women have come” so they know- health workers, that these need to be attended to. So it’s really a good intervention from manifest project. In fact we appreciate if they – manifest could come another round atleast it would be good”.* **Community Development officer, Key informant interview, Pallisa District**
