# Supplementary material for: Participatory monitoring and evaluation approaches that influence decision-making: lessons from a maternal and newborn study in Eastern Uganda
Source: Health Res Policy Syst. 2017 Dec 28;15(Suppl 2):107. doi: 10.1186/s12961-017-0274-9 (PMC5751403; doi:10.1186/s12961-017-0274-9)
Supplement: Supplementary file 2 — Data use for decision-making story of change. (DOC 30 kb) [file 12961_2017_274_MOESM2_ESM.doc]

*“…..as a result of MANIFEST study, we now use a lot of our data in planning and budgeting. For example, not long ago we did not have adequate resources to construct maternity wards in every Sub-county, so we had to use our data and we said okay, which place has the biggest ANC attendances, deliveries, which place has big out-patient attendances. We then decided that we have the general ward constructed in Kadama health center III, in Kadama Sub-county. So, we are now using our data because it is now available contrary to what was there before, where you would ask ahh… how many deliveries do you have on average per month and you’re like aaa---- oba this number [guessing]. But now we can easily check, all the health indicators because we have a data center where all our information is readily available. So, we can use our data for planning and decision-making, and even staff allocation. For example, we decided to allocate more midwives and other health workers to facilities that have high numbers of ANCs/deliveries and outpatients respectively. In addition, we have used this information to justify the need for more health workers, which has convinced the Ministry of health to consider relaxing the ban on the recruitment of health workers.* ***District Health Officer***
