# Supplementary material for: Participatory monitoring and evaluation approaches that influence decision-making: lessons from a maternal and newborn study in Eastern Uganda
Source: Health Res Policy Syst. 2017 Dec 28;15(Suppl 2):107. doi: 10.1186/s12961-017-0274-9 (PMC5751403; doi:10.1186/s12961-017-0274-9)
Supplement: Supplementary file 3 — Changes management and leadership story of change. (DOC 26 kb) [file 12961_2017_274_MOESM3_ESM.doc]

*“Then as administrators through the trainings, like personally I was trained in health service management so somehow, I realized that, my skills have improved for example the way I manage the other staff, the way I plan for the health unit. Yes, and the way I account for resources at the facility. At the facility we now have regular meetings, we have the staff meetings, and we have the health unit management meetings, which we used not to have on a regular basis. Previously when it came to the health management meetings, I would not involve many stakeholders who are relevant when it came to plan for the facility. I would concentrate around the health unit management committee members but these days there are other stakeholders whom I invite like politicians at the sub county or at the district, all the administrators and sometimes when we discuss certain issues in their presence, diverse ideas are accepted (bought) easily when they come and attend the meeting. For instance, for the renovation of theater that is going on previously it was not easy to convince them but after involving them, when they came on board they were able to convince the district to take up that role and now the theater is being renovated.*

*The staff meetings are now more regular because they are now carried out on a monthly basis and when we conduct these meetings also, what used to happen is we would just meet without any objectives. But now days we meet to discuss certain issues so somehow you realize that we have come up with action plans which are somewhat realistic. For example, we may decide to review our performance and identify where the gaps are and then as staff in our meeting we decide on the way forward. Before we were a little bit disorganized without clearly stated objectives and the meetings were even irregular.*

*Also, what used to happen is that you would sit as the in charge and draw your own work plan and you spend the way you liked, now it’s a bit more inclusive. The staffs are engaged in the planning process, the management is engaged, and you find that when you make a work plan it’s representative of the needs that are at the facility”.* ***In charge Health center IV, Kamuli District***
